# Supplementary material for: Neighborhood Socioeconomic Resources and Crime-Related Psychosocial Hazards, Stroke Risk, and Cognition in Older Adults
Source: Int J Environ Res Public Health. 2021 May 12;18(10):5122. doi: 10.3390/ijerph18105122 (PMC8151671; doi:10.3390/ijerph18105122)
Supplement: Supplementary file 1 [file ijerph-18-05122-s001.zip › ijerph-1180578-supplementary.pdf]

Supplemental Table S1. Pearson correlations of socioeconomic resources and FSRP-10

	FSRP-10
% below poverty level	-0.08, p=0.42
Median household income	0.08, p=0.43
% with less than 12 years of education	-0.16, p=0.09
% with 16+ years of education	0.22, p=0.02
% unemployment	-0.07, p=0.45

Abbreviations: FSRP-10 = Framingham Stroke Risk Profile score 10-year risk of stroke; % = percent.
